# Inhibition of Hepatitis C Virus 3a genotype entry through Glanthus Nivalis Agglutinin

**DOI:** 10.1186/1743-422X-8-248

**Published:** 2011-05-20

**Authors:** Usman A Ashfaq, Muhammad S Masoud, Saba Khaliq, Zafar Nawaz, Sheikh Riazuddin

**Affiliations:** 1 Division of Molecular Medicine, Centre of Excellence in Molecular Biology, University of the Punjab, Lahore, Pakistan; 2 Braman Family Breast Cancer Institute, University of Miami, USA; 3 Allama Iqbal Medical College, Allama Shabir Ahmad Usmani Road, Lahore, Pakistan

## Abstract

**Background:**

Hepatitis C Virus (HCV) has two envelop proteins E1 and E2 which is highly glycosylated and play an important role in cell entry. Inhibition of virus at entry step is an important target to find antiviral drugs against HCV. Glanthus Nivalis Agglutinin (GNA) is a mannose binding lectin which has tendency for specific recognition and reversible binding to the sugar moieties of a wide variety of glycoproteins of enveloped viruses.

**Results:**

In the present study, HCV pseudoparticles (HCVpp) for genotype 3a were produced to investigate the ability of GNA to block the HCV entry. The results demonstrated that GNA inhibit the infectivity of HCVpp and HCV infected serum in a dose-dependent manner and resulted in 50% reduction of virus at 1 ± 2 μg concentration. Molecular docking of GNA and HCV glycoproteins (E1 and E2) showed that GNA inhibit HCV entry by binding N-linked glycans.

**Conclusion:**

These results demonstrated that targeting the HCV glycans is a new approach to develop antiviral drugs against HCV.

## Background

HCV is a serious health problem that affects 200 million people worldwide and 10 million people in Pakistan [1]. HCV causes acute and chronic hepatitis infection which can eventually lead to permanent liver damage, hepatocellular carcinoma and death [2]. It was estimated by the World Health Organization in 2004 that the annual deaths due to liver cancer caused by HCV and cirrhosis were 308,000 and 785,000, respectively [3]. HCV is a blood-borne pathogen, which transmitted through parenteral exposure to contaminated blood or body fluids [4]. Factors most strongly associated with HCV infection are blood transfusion (56%), alcohol consumption (44%) and intravenous drug abuse (31%) [4,5]. Some other risk factors include use of inadequately sterilized medical equipment, high-risk sexual behaviors, and social or cultural practices such as body piercing, circumcision, and tattooing [6,7].

HCV is a small enveloped virus with a positive-sense, single-stranded RNA genome that encodes a large polyprotein of 3010 amino acids (aa). The polyprotein is co- and post-translationally processed by cellular and virally encoded proteases to produce four structural (Core, E1, E2 and P7) and six non-structural proteins (NS2, NS3, NS4A, NS4B, NS5A, NS5B). HCV envelop proteins E1 and E2 are highly glycosylated and play an important role in cell entry. E1 serves as the fusogenic subunit and that E2 acts as the receptor binding subunit of the HCV envelope. The E1 envelope glycoprotein of HCV contains 4 to 5 N-linked glycans and the E2 envelope glycoprotein has 11 N-glycosylation sites [8,9]. However, the number of glycosylation sites varies according to genotype. Glycosylation sites on E1 and E2 are highly conserved and consists a combination of complex and high-mannose side-chains. HCV glycans play an important role in envelope glycoprotein folding and formation of the HCV E1E2 complexes, receptor interactions, virus entry [9] and antigenic variation [10].

Galanthus nivalis agglutinin (GNA), known as snowdrop lectin is a mannose-specific, tetrameric protein, consisting of 4 identical subunits of approximately 12 kDa [11]. GNA is carbohydrate binding agent which has tendency for specific recognition and reversible binding to the sugar moieties of a wide variety of glycoproteins. GNA directly interact with the glycans of the viral glycoprotein of enveloped viruses and causes interruption of virus entry (i.e. fusion) into its target cell [12-14].

In the current study, HCV entry is blocked by binding the Glanthus Nivalis Agglutinin with HCV glycosylation site. For this purpose first of all HCV E1 and E2 envelope proteins of local 3a genotype were docked with GNA to find out the target site of ligand binding. Then toxicity of GNA in liver and fibroblast cells was checked though trypan blue dye and MTT cell proliferation assay. After the toxicological analysis of GNA, HCV entry was found to be blocked at non toxic dose.

## Materials and methods

### Sample Collection

Sera from patients chronically infected with HCV without any previous history of antiviral treatment were collected from Molecular Diagnostic Lab, Centre of Excellence in Molecular Biology (CEMB) under the provision of Institutional Review Board (IRB) of CEMB. The participating subjects gave informed consent for the collection of blood samples for this study. The estimated duration of infection varied from 6 months to 10 years among the patients. Study included HCV blood from both male and female patients excluding children. The diagnosis of chronic HCV was based on elevated serum ALT (SGPT) and AST (SGOT) levels at least for six months, histological examination, and consistent detection of serum HCV RNA were present in each patient. All patients were negative for HBs Ag.

### Cell lines

Huh-7 and HEK 293 T cells were cultured in Dulbecco's Modified Eagle medium (DMEM) supplemented with 10% fetal calf serum, 100 IU/ml penicillin and 100 μg/ml streptomycin, at 37°C in an atmosphere of 5% CO_2_. CHO cells were cultured in DMEM Hams F12 supplemented with 5% fetal calf serum, 100 IU/ml penicillin and 100 μg/ml streptomycin. Huh-7 was kindly provided by Dr. Zafar Nawaz (Biochemistry and Molecular Biology Department, University of Miami, USA). CHO was provided by Dr. Ahmad Usman Zafar (Biopharmaceutical Lab, CEMB, Pakistan).

### Plasmids

The pcDNA-E1E2 expression vector encoding the E1 and E2 glycoproteins (171-746) of HCV genotype 3a, was generated by inserting into a nonpackageable, CMV promoter-driven expression construct. (provided by Shazia Rafique, virology lab, CEMB, Pakistan). The CMV-Gag-Pol murine leukemia virus (MLV) packaging construct, encoding the MLV *gag *and *pol *genes, and the pTG-Luciferase plasmid provided by Dr. Jaean Dubison, France.

### Chemicals

Glanthus Nivalis Agglutinin was purchased from Sigma Aldrich (Cat. No.L8275 St. Louis, MO, USA. Monoclonal antibody specific to HCV E2 (Cat. No.sc-57769 was purchased from Santa Cruiz Biotechnology. Glyceraldehydes-3-phosphate dehydrogenase (GAPDH) and secondary goat anti-mouse monoclonal antibodies were purchased from Sigma Aldrich (St. Louis, MO, USA).

### Molecular docking of GNA with HCV E1 and E2 glycoprotein

Molecular docking is a method which predicts the preferred orientation of one molecule to a second when bound to each other to form a stable complex [15]. Docking is frequently used to find out the binding orientation of small molecule drug candidates to their protein targets in order to in turn predict the affinity and activity of the small molecule. Hence, docking plays an important role in drug design [15].

The 3-D structures of GNA lectin were obtained from Swiss Prot (PDB code: 1JPC, 1NIV) (http://www.rcsb.org/pdb/home/home.do), (Acc. no: 1JPC, 1NIV). 1JPC structure is a complex of mannose specific agglutinin (lectin) from snow drop (*Galanthus *Nivalis) bulbs with mannose-alpha1, 6-(mannose-alpha1, 3)-mannose-alpha1, 6-(mannose-alpha1, 3)-mannose. 1NIV structure is a complex of mannose specific agglutinin (lectin) from snow drop (*Galanthus Nivalis*) bulbs with mannose-alpha1, 3-methyl-D-mannose. For 3-D structure of HCV protein, sequence of HCV was taken from EMBL (http://www.ebi.ac.uk/, Acc. No: ABY83295, M67463). 3-D protein model of HCV was generated from MUSTER (http://zhang.bioinformatics.ku.edu/). Vakser server was used for protein-protein docking (http://vakser.bioinformatics.ku.edu/). Ten protein-protein complexes were generated for each 3-D model by protein docking through vakser.

### Production of HCVpp and infection

HCVpp were produced by co-transfection of 293-T cells with equal amounts of three expression vector as described previously [16]. Supernatants containing pseudoparticles were harvested 48 h later, filtered through 0.45 μm pore-sized membranes and stored at -80°C before use in infection of Huh7 cells.

### Cell proliferation assay

MTT (3-[4, 5-dimethylthiazol-2-yl]-2, 5-diphenyltetrazolium bromide) is a rapid and sensitive *in-vitro *procedure for evaluating cellular toxicity of compounds. The MTT substance is reduced by mitochondrial succinic dehydrogenases in living cells to purple formazan crystals that are not soluble in aqueous water. The absorption of dissolved formazan in the visible region correlates with the number of viable cells [17]. To investigate cellular toxicity, 2 × 10^4 ^Huh-7 cells were plated into 96-well plates. After 24 h, different concentrations of GNA were added and the plate was sealed and kept at 37°C in an atmosphere of 5% CO_2 _for 24 h. After 24 h, fresh media (100 μl) and MTT solution (5 mg/ml in PBS) were added to all wells in Columns 1-11. Wrapped the plate in aluminium foil and incubated for 3-4 h at 37°C. Media was carefully removed and added 100 μl of DMSO to dissolve the formazan crystals in Columns 1-11. MTT formazan product was determined by measuring absorbance with an enzyme-linked immunosorbent assay (ELISA) plate reader at a test wavelength of 570 nm and a reference wavelength of 620 nm.

Cell viability was obtained using the following equation.

### Anti-infectivity of GNA in liver cells

Huh-7 cells were seeded at density of 3 × 10^5 ^cells per 35 mm plate. After 24 h, cells were washed twice with 1XPBS. Then, viral inoculations of 10^5 ^IU HCV virus of genotype 3a in each well were performed after washing twice with 1X PBS. First well was considered as control (only HCV infected serum and solvent in which GNA was dissolved) and added dose of GNA (minimum cell killing) in the remaining five wells on the same day for checking the antiviral response of compounds. Total RNA was isolated by using Gentra RNA isolation kit (Gentra System Pennsylvania, USA) according to the manufacturer's instructions. Briefly, cells were lysed with cell lysis solution containing 5 μl internal control (Sacace Biotechnologies Caserta, Italy). RNA pallet was solubilized in 1% DEPC (Diethyl pyrocarbonate treated water). HCV RNA quantifications were determined by Real Time PCR Smart Cycler II system (Cepheid Sunnyvale, USA) using the Sacace HCV quantitative analysis kit (Sacace Biotechnologies Caserta, Italy) according to the manufacturer's instructions.

### Formula for the calculation of HCV RNA concentration

Following formula was used to calculate the concentration HCV RNA of each sample.

IC = internal control, which is specific for each lot.

### Protein isolation and estimation

Cells were harvested after the 24 h of transfection and protein was isolated for expression analysis. Transfected cells were washed with 1X PBS. TEN buffer at volume of 500 μl was added to dislodge the cells and then were scrapped off after 15 sec. The cells were then pellet down by centrifugation for 5 min at 13000 rpm at 4°C. Pellet down cells were then lysed by adding 100 μl of lysis buffer (50 mM Tris-Cl, pH 8.0, 150 mM NaCl, 0.02% Sodium azide, 1% Triton X-100, 1 μg/ml protease inhibitors, and 100 μg/ml PMSF), incubated on ice for 15 min and centrifuged at 13,000 rpm at 4°C for 30 min. Supernatant containing protein was stored at -20°C. The isolated protein was quantified by spectrophotometric method. Protein sample (1 μl) was diluted in 800 μl of 1X PBS and 200 μl of Bio Rad dye and absorption was taken at 595 nm of wavelength.

### Western blotting

Western blotting provides information about presence, molecular weight, and/or quantity of an antigen by combining protein separation via gel electrophoresis with specific recognition of antigens by antibodies. To study protein expression of HCV genes and inhibitory effect of compounds, 100 μg of total protein was loaded on 10% SDS-PAGE gels and electrophoretically blotted onto a nitrocellulose membrane (Bio-Rad). The membranes were blocked for 1 h at room temperature with phosphate-buffered saline containg 5% skim milk and incubated with HCV specific primary antibody. After being washed with 1X PBS containing 0.1% Tween 20, the membranes were treated with monoclonal antibodies specific to HCV envelop protein E2 and GAPDH (Santa Cruz Biotechnology). The membrane was then treated with HRP conjugated anti mouse secondary antibody for 1 h at room temperature. The expression of protein was evaluated by using chemiluminescence's detection kit (Sigma Aldrich).

### Statistical Analysis

All statistical analysis was done using SPSS software (version 16.0, SPSS Inc). Data are presented as mean ± SD. Numerical data were analyzed using student's t-test and ANOVA. P value < 0.05 was considered statistically significant.

## Results

### Docking of mannose binding against HCV glycoprotein

**Figure 1:**
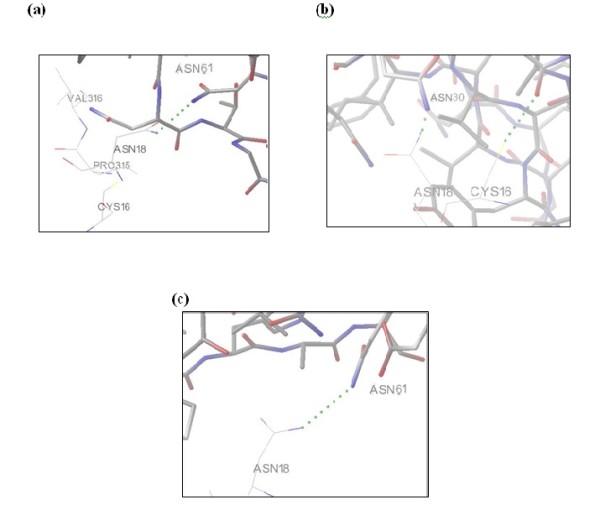
**Docking of GNA lectin with HCV glycoprotein**. (a) Interaction of Asn18 of HCV with Asn61 of 1JPC GNA lectin. (b) Interaction of Asn18 of HCV with Asn30 of 1JPC GNA lectin. (c) Interaction of Asn18 of HCV with Asn61 of 1NIV GNA lectin

Before docking, binding sites for GNA was observed. The residues involved in binding with mannose in all three 3D structures of GNA were Gln26, Asp28, Asn30, Tyr34, Gln57, Asp59, Asn61, Tyr65, Gln89, Asp91, Asn93 and Tyr97. N-glycosylation sites for HCV protein were also examined by carrying out pair alignment of sequences of HCV subtype 3a (Acc. No: ABY83295) with HCV subtype 1a (Acc. No: M67463) (Data not shown). Interactions among different residues, in each of the ten protein-protein complexes of both 3-D structures were observed. Among these the most interesting interactions are the binding of active site residues of GNA lectin Asn30 and Asn61 with N glycosylation site of HCV, Asn18. The interaction of Asn 18 of HCV protein with Asn30 and Asn61 of GNA lectin was observed in protein-protein complex of 1JPC, whereas in 1NIV interaction of only Asn18 with Asn61 was observed. Figure 1 shows that HCV protein does show interactions with GNA lectin. Some of the residues show interaction with binding pockets of GNA lectin. Interactions among the N-glycosylation site of HCV and binding pocket of GNA lectin was also observed. From these results it can be concluded that GNA lectin may play role in inhibiting HCV activity and hence can be considered for further studies.

### Toxicological study of GNA in liver and fibroblast cells

**Figure 2:**
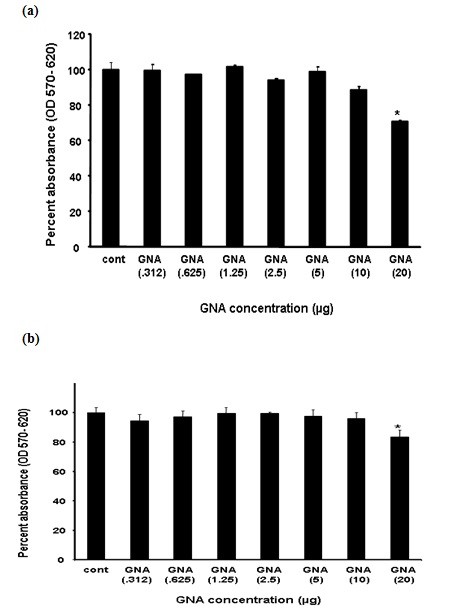
**Toxicological study of GNA in Huh-7 and CHO cell**: Huh-7 cells were plated at the density of 2 × 10^4^in 96 well plates. After 24 h cells were treated with different concentrations of GNA and control consisted of solvent in which GNA dissolved. After 24 h incubation period add MTT solution to all wells and incubated for 3-4 h at 37°C. Viable cells convert MTT to purple formazan crystal. Added DMSO to dissolve the formazan crystals and read absorbance at 570 nm and 620 nm. (a) Toxicological analysis of GNA in Huh-7 cells through MTT cell proliferation assay. (b) Toxicological analysis of GNA in CHO cells through MTT cell proliferation assay.

Cytotoxic effects of GNA were analyzed after 24 h incubation of Huh-7 and CHO cells with different concentration of GNA. Figure 2 depicts cytotoxicity analysis of GNA and demonstrates that Huh7 and CHO cell viability is unaffected by concentrations up to 10 μg. However, when exceed from 10 μg, toxic effect in liver and fibroblast cells has been observed. The data verified by microscopic examination of cells and standard trypan blue dye measurement, which demonstrate GNA has no toxic effect at 10 μg concentration.

### Anti infectivity of GNA against HCVpp

**Figure 3:**
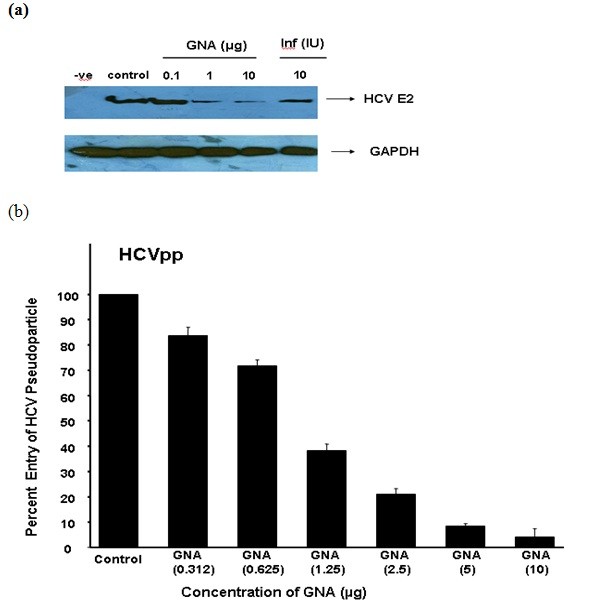
**Antiviral effect of GNA against HCVpp in liver cells**. HCVpp was produced in HEK 293 T cells and collected in media after filtration in 0.45 micron filter. (a) GNA was incubated with HCV pseudo particle at 37°C for 1 hr. After 1 hrs Huh-7 cells were infected with pseudo particle of HCV 3a genotype in the presence and absence of GNA per well and incubated for additional 48 hrs. At the end of incubation period protein were isolated and analyzed by western blotting with anti E2 monoclonal antibody and GAPDH serve as internal control. (b) GNA was incubated with HCV pseudo particle at 37°C for 1 h. After 1 h Huh-7 cells were infected with pseudo particle of HCV 3a genotype in the presence and absence of different concentrations of GNA and incubated for 3 hrs. After 24 h cells were lysed and luciferase activity was determined by using a luminometer. Luciferase activity is not reported as an absolute value, but is calculated relative to the 'no drug' condition and reported on the *y*-axis as a percentage. Results are represented as the average and standard error for three independent experiments. P value > 0.05 vs control was considered as statistically significant.

**Figure 4:**
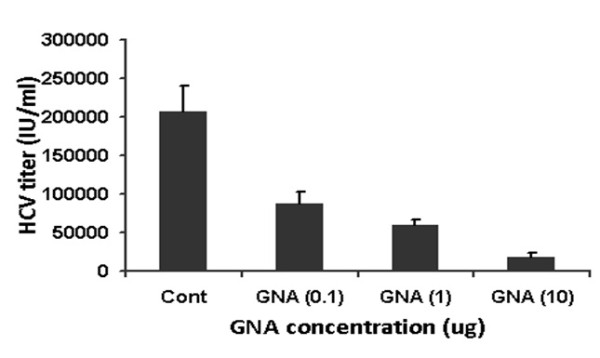
**Antiviral effect of GNA against HCV 3a genotype in liver cells**. GNA was incubated with 2 × 10^5 ^HCV 3a virus at 37°C for I h. After 1 h cells were infected with 2 × 10^5 ^copies of HCV 3a genotype per well and incubated for additional 24 h. At the end of incubation period, total RNA was extracted by Gentra kit, and the levels of HCV RNA remaining were determined, by real time Quantitative RT-PCR assay and are shown as percentage of HCV RNA survival in cells. Results are represented as the average and standard error for three independent experiments. P value > 0.05 vs control was considered as statistically significant.

To determine the effect of GNA on HCV infection, HCVpp are produced by transfecting the three vectors in Human embryo kidney cells (293T). The first vector encodes retroviral Gag and Pol, second vector encodes a reporter protein Luciferase and third vector encodes HCV glycoproteins E1 and E2. 293T cells secrete virus pseudo particle an average 10^5 ^particle/ml and collect in media after filtration of 0.45 micron filter. These pseudo particles can be used to infect Huh-7 cells and infectivity is evaluated by quantification of amount of luciferase and through western blotting with HCV E2 specific monoclonal antibody in the presence and absence of GNA. Figure 3a and 3b demonstrates that GNA has antiviral effect against HCVpp in dose-dependent manner. Figure 4 exhibited that GNA showed 50% (EC 50) reduction of virus at 1 μg concentration. GNA exhibited 90% (EC 90) reduction of virus at 5 μg concentration. GNA showed antiviral effect by binding to HCV glycoprotein. These results indicate that GNA possesses anti-HCV activity at a concentration that does not affect cell growth.

## Discussion

Viral entry is an attractive target to find out antiviral drugs against HCV. A major advancement to look into HCV entry process was the development of HCVpp, consisting of native HCV envelope glycoproteins, E1 and E2, assembled onto retroviral core particles [8,16,18]. This system is potentially very powerful tool to identify and characterize molecules that block HCV entry. In this study, HCVpp of local HCV genotype 3a were produced to study early entry steps mediated by HCV envelope glycoproteins. This assay is based on the quantification of retroviral DNA synthesis, which occurs soon after the fusion of the retroviral particle with a cellular membrane. Presumably, this assay is only dependent on the entry steps mediated by the heterodimer E1E2 (binding, endocytosis, and fusion) and on the activity of the reverse transcriptase of the HCVpp retroviral core. Furthermore, data obtained with HCVpp was also confirmed with the infection of whole virus of HCV genotype 3a in liver cells.

HCV E1 envelope glycoprotein contain 4 to 5 N-linked glycosylation sites and the E2 envelope glycoprotein has 11 N-glycosylation sites [8,19,20]. These N-linked glycosylation sites play an important function in entry, folding and modulating the immune response. GNA lectin has been shown antiviral activities against many enveloped viruses by blocking entry such as HIV [21,22], Simian immunodeficiency virus (SIV)[23], HCMV and influenza virus. Our data verified that GNA inhibit HCVpp and HCV whole genome entry in a dose-dependent manner and resulted in 50% reduction of virus at 1 ± 2 μg concentration (Figure 3a and 3b). HCV serum particles were used as positive control to study the antiviral effect on real HCV particles (Figure 4). Molecular docking results showed that GNA inhibit HCV entry by binding to N-linked glycans. However, due to the absence of a 3D structure for HCV envelope glycoproteins, it is difficult to identify spatial relationship between HCV glycans and the CD81 binding site. Contrary to HIV, where mutation in glycosylation sites resulted in the resistance against GNA [14,24], but the glycosylation sites in HCV are strongly conserved minimizing the chance of creating resistance against such drugs targeting glycans [25]. These data clearly confirmed that targeting HCV envelope glycans might be a promising approach in the development of novel antiviral therapies.

Development of inhibitors of virus entry, such as GNA, will reinforce the arsenal against HCV. The current treatment of standard interferon in combination with ribavirin, has limited benefits due to emergence of resistant mutations during long-term treatment, adverse side effects and high cost. Consequently, there is a dire need for the development of more effective, less toxic antiviral agents. With the passage of time resolution of the three-dimensional structure of various HCV components and the development of *in-vitro *assays such as HCVpp and HCVcc to assess the antiviral potency of molecules targeting these elements have made it possible to screen and develop specific HCV inhibitors [26]. Currently, the major targets of new antiviral are the HCV internal ribosome entry site, NS3 serine protease, and NS5B, the RNA-dependent RNA polymerase. Although a small number of compounds have started to show promising results in early-phase clinical trials, preclinical evidence is accumulating, demonstrating that development of resistance will eventually limit the efficacy of these new drugs [27]. This is due to the fact that HCV is a highly variable virus with rapid viral kinetics, large population sizes, and a quasispecies distribution [27]. Therefore, combinations of multiple drugs with different targets will be required to treat chronic HCV. Our study demonstrated that GNA inhibit HCV entry by binding to N-linked glycan at non toxic concentration. These studies showed that HCV entry process will help to provide new inhibitors such as GNA and combination of entry inhibitors with NS3 protease and NS5b RNA dependent polymerase will provide better option to treat chronic HCV.

## Abbreviations

**HCV**: Hepatitis C virus; **Huh-7**: Human Hepatoma Cell line. **HCVpp**: HCV pseudoparticles.

## Competing interests

The authors declare that they have no competing interests.

## Authors' contributions

UAA performed lab work and manuscript write up. MSM, SK, ZN and SRD helped me in writing the manuscript. All the authors read and approved the final manuscript.

## Authors' information

Usman Ali Ashfaq (PhD Molecular Biology), Muhammad Shareef Masoud (PhD Molecular Biology), Saba Khaliq (PhD Molecular Biology), Sheikh Riazuddin (PhD molecular Biology and Dean Post graduate study at Allama Iqbal medical college, Lahore

## References

[B1] Raja NS Janjua NK Epidemiology of hepatitis C virus infection in Pakistan. J Microbiol Immunol Infect J Microbiol Immunol Infect 2008 41 4 8 18327420

[B2] Berenguer M Lopez-Labrador FX Wright TL Hepatitis C and liver transplantation J Hepatol 2001 35 666 678 10.1016/S0168-8278(01)00179-9 11690716

[B3] WHO: World Health Organization Department of Measurement and Health Information 2004 http://www.who.int/healthinfo/statistics/bodgbddeathdalyestimates.xls

[B4] Scannell KM Willard CC Seeff LB National Institutes of Health Consensus Development Conference Statement: management of hepatitis C Hepatology 2002 36 S3 S20 12407572 10.1053/jhep.2002.37117

[B5] Barazani Y Hiatt JR Tong MJ Busuttil RW Chronic viral hepatitis and hepatocellular carcinoma World J Surg 2007 31 1243 1248 17440771 10.1007/s00268-007-9041-3

[B6] Saleem NH Adrien A Razaque A Risky sexual behavior, knowledge of sexually transmitted infections and treatment utilization among a vulnerable population in Rawalpindi, Pakistan Southeast Asian J Trop Med Public Health 2008 39 642 648 19058600

[B7] Karmochkine M Carrat F Dos Santos O Cacoub P Raguin G A case-control study of risk factors for hepatitis C infection in patients with unexplained routes of infection J Viral Hepat 2006 13 775 782 10.1111/j.1365-2893.2006.00742.x 17052278

[B8] Drummer HE Maerz A Poumbourios P Cell surface expression of functional hepatitis C virus E1 and E2 glycoproteins FEBS Lett 2003 546 385 390 10.1016/S0014-5793(03)00635-5 12832074

[B9] Goffard A Callens N Bartosch B Wychowski C Cosset FL Montpellier C Dubuisson J Role of N-linked glycans in the functions of hepatitis C virus envelope glycoproteins J Virol 2005 79 8400 8409 10.1128/JVI.79.13.8400-8409.2005 15956584 PMC1143753

[B10] Slater-Handshy T Droll DA Fan X Di Bisceglie AM Chambers TJ HCV E2 glycoprotein: mutagenesis of N-linked glycosylation sites and its effects on E2 expression and processing Virology 2004 319 36 48 10.1016/j.virol.2003.10.008 14967486

[B11] Van Damme EJM Allen AK Peumans WJ Isolation and characterization of lectin with exclusive specificity towards mannose from snowdrop (Galanthus nivalis) bulbs FEBS Lett 1987 215 140 144 10.1016/0014-5793(87)80129-1

[B12] Balzarini J Schols D Neyts J Van Damme E Peumans W De Clercq E Alpha-(1-3)- and alpha-(1-6)-D-mannose-specific plant lectins are markedly inhibitory to human immunodeficiency virus and cytomegalovirus infections in vitro Antimicrob Agents Chemother 1991 35 410 416 1645507 10.1128/aac.35.3.410 PMC245024

[B13] Balzarini J Neyts J Schols D Hosoya M Van Damme E Peumans W De Clercq E The mannose-specific plant lectins from Cymbidium hybrid and Epipactis helleborine and the (N-acetylglucosamine)n-specific plant lectin from Urtica dioica are potent and selective inhibitors of human immunodeficiency virus and cytomegalovirus replication in vitro Antiviral Res 1992 18 191 207 10.1016/0166-3542(92)90038-7 1329650

[B14] Balzarini J Hatse S Vermeire K Princen K Aquaro S Perno CF De Clercq E Egberink H Vanden Mooter G Peumans W Mannose-specific plant lectins from the Amaryllidaceae family qualify as efficient microbicides for prevention of human immunodeficiency virus infection Antimicrob Agents Chemother 2004 48 3858 3870 10.1128/AAC.48.10.3858-3870.2004 15388446 PMC521907

[B15] Lengauer T Rarey M Computational methods for biomolecular docking Curr Opin Struct Biol 1996 6 402 406 10.1016/S0959-440X(96)80061-3 8804827

[B16] Bartosch B Dubuisson J Cosset FL Infectious hepatitis C virus pseudo-particles containing functional E1-E2 envelope protein complexes J Exp Med 2003 197 633 642 10.1084/jem.20021756 12615904 PMC2193821

[B17] Mosmann T Rapid colorimetric assay for cellular growth and survival: application to proliferation and cytotoxicity assays J Immunol Methods 1983 65 55 63 10.1016/0022-1759(83)90303-4 6606682

[B18] Hsu M Zhang J Flint M Logvinoff C Cheng-Mayer C Rice CM McKeating JA Hepatitis C virus glycoproteins mediate pH-dependent cell entry of pseudotyped retroviral particles Proc Natl Acad Sci USA 2003 100 7271 7276 10.1073/pnas.0832180100 12761383 PMC165865

[B19] Voisset C Dubuisson J Functional hepatitis C virus envelope glycoproteins Biol Cell 2004 96 413 420 10.1016/j.biolcel.2004.03.008 15325070

[B20] Meunier JC Fournillier A Choukhi A Cahour A Cocquerel L Dubuisson J Wychowski C Analysis of the glycosylation sites of hepatitis C virus (HCV) glycoprotein E1 and the influence of E1 glycans on the formation of the HCV glycoprotein complex J Gen Virol 1999 80 Pt 4 887 896 10211957 10.1099/0022-1317-80-4-887

[B21] Balzarini J Van Damme L Microbicide drug candidates to prevent HIV infection Lancet 2007 369 787 797 10.1016/S0140-6736(07)60202-5 17336656

[B22] Pollicita M Schols D Aquaro S Peumans WJ Van Damme EJ Perno CF Balzarini J Carbohydrate-binding agents (CBAs) inhibit HIV-1 infection in human primary monocyte-derived macrophages (MDMs) and efficiently prevent MDM-directed viral capture and subsequent transmission to CD4+ T lymphocytes Virology 2008 370 382 391 10.1016/j.virol.2007.08.033 17928023

[B23] Francois KO Auwerx J Schols D Balzarini J Simian immunodeficiency virus is susceptible to inhibition by carbohydrate-binding agents in a manner similar to that of HIV: implications for further preclinical drug development Mol Pharmacol 2008 74 330 337 10.1124/mol.108.047621 18474667

[B24] Witvrouw M Fikkert V Hantson A Pannecouque C O'Keefe B R McMahon J Stamatatos L de Clercq E Bolmstedt A Resistance of human immunodeficiency virus type 1 to the high-mannose binding agents cyanovirin N and concanavalin A J Virol 2005 79 7777 7784 10.1128/JVI.79.12.7777-7784.2005 15919930 PMC1143621

[B25] Goffard A Dubuisson J Glycosylation of hepatitis C virus envelope proteins Biochimie 2003 85 295 301 10.1016/S0300-9084(03)00004-X 12770768

[B26] De Francesco R Migliaccio G Challenges and successes in developing new therapies for hepatitis C Nature 2005 436 953 960 10.1038/nature04080 16107835

[B27] Pawlotsky JM Therapy of hepatitis C: from empiricism to eradication Hepatology 2006 43 S207 220 10.1002/hep.21064 16447262

